# Response of Transitional Mixtures Retaining Memory of In-Situ Overburden Pressure Monitored Using Electromagnetic and Piezo Crystal Sensors

**DOI:** 10.3390/s21072570

**Published:** 2021-04-06

**Authors:** Sang Yeob Kim, Jong-Sub Lee, Junghee Park

**Affiliations:** 1 Department of Civil and Environmental Engineering, University of Illinois at Urbana-Champaign, 205, North Mathews Avenue, Urbana, IL 61801, USA; s3778@illinois.edu; 2 School of Civil, Environmental and Architectural Engineering, Korea University, 145, Anam-ro, Seongbuk-gu, Seoul 02841, Korea; jongsub@korea.ac.kr

**Keywords:** engineering properties, revised soil classification system, soil mixtures, threshold fines fraction

## Abstract

The major and minor components in granular soil materials determine their properties and behavior. This study explores the transitional behavior within threshold fines fraction of soil mixtures based on the data from the literature and experiments. From the literature survey, the void ratio, shear wave velocity, compression index, and friction angle capture the transitional turning point between the low and data-adjusted high threshold fines fractions. In particular, there is a dramatic change in hydraulic conductivity below the low threshold fines fraction that highlights the critical role of small amounts of fines in the fluid flow (e.g., clogging). From an experimental study, the engineering properties of natural soil samples identified using deformation and elastic wave sensors show transitional trends within the Revised Soil Classification System framework. The evolution of compressibility and shear wave velocity indicate that either coarse, fine, or both particles are likely to contribute to large and small strain stiffnesses when the effective stress is below 400 kPa. Thereafter, both engineering properties indicate that the soil sample retains a memory of in-situ overburden pressure when the effective stress is around 400 kPa. There is a critical role of fines that are slightly higher than low threshold fines fraction on engineering properties that promote the application of Revised Soil Classification System RSCS to natural soils.

## 1. Introduction

Soils are granular materials and contain a broad range of sizes. The particle size determines the participation rate of particles in the soil matrix and controls their engineering behavior [1]. Furthermore, the particle size distribution is a good indicator that represents the particle-level forces, inter-particle packing, and macroscale behavior [1,2,3,4]. Most notably, particle scale analyses indicate that particle size determines the governing force in granular materials. For example, capillary and electrical forces prevail when the grain size is less than 75 µm; otherwise, skeletal and weight forces become more dominant at large grain sizes [5].

Therefore, geotechnical engineering often uses the 75 µm of grain size criteria based on 50% boundary by weight to divide soils into coarse-grained or fine-grained soil for engineering purposes [6]. However, soils are originally a mixture of particles; therefore, both the major and minor components in soil mixtures control their properties and behavior [7,8,9]. In this context, previous studies investigated the role of fines on engineering soil properties, revealing that finer particles as a minor component play a critical role in determining mechanical response and fluid flow in soil mixtures classified as even coarse-grained soils [10,11,12,13,14,15,16]. Furthermore, there are significant changes in engineering soil properties at the threshold fines fraction. Related phenomena include changes in void ratio, small strain stiffness, compression index, friction angle, and hydraulic conductivity as a function of the fines fraction [17,18,19,20,21].

In particular, the threshold fines fractions are even lower than 50% by weight and vary with soil index properties such as particle shape, coefficient of uniformity, and liquid limit of fines when passing Sieve No. 200 (corresponding particle size = 0.075 mm). The soil-specific threshold fines fractions combined with physics-inspired and data-driven analyses accumulated in a laboratory during the last century led to the development of the Revised Soil Classification System (RSCS) [22,23]. However, the application of RSCS to natural soils (or well-graded mixtures) remains a challenge.

This study examines the soil-specific threshold fines fraction and the transition from coarse- to fine-controlled engineering behavior of soil mixtures based on data extracted from the literature. Further, a soil sample obtained from the Savannah River Site is characterized using physical sensors (i.e., deformation and elastic wave sensors) in the context of the void ratio, compressibility, and small strain stiffness. The experimental results are then analyzed within the RSCS framework. The tested soil sample classified as a transitional mixture in the RSCS reinforces that either fine, coarse, or both fractions can control the mechanical response. This study begins with volumetric-gravimetric analyses to introduce the low- and high-threshold fines fractions combined with soil property data.

## 2. Theoretical Aspects

### 2.1. Low- and High-Threshold Fine Fractions

This section defines the threshold fine fraction *F_th_* based on gravimetric-volumetric relations of a binary mixture (i.e., coarse and fine grains) assuming that the characteristic grain size of the fine components is much smaller than that of the coarse components, and both the fine and coarse components can experience extremely dense or loose packing. This approach is practical because the boundaries developed by the coarse grains influence the packing conditions of fine grains [24]. Furthermore, coarse-grained sediments can form a load-carrying skeleton, although the void ratio of coarse grains exceeds its maximum void ratio (i.e., *e_C_* > *e^max^*) as fines improve the stability of the soil matrix and hinder the buckling of chains made of coarse grains [21,25]. In this context, gravimetric-volumetric relations lead to the definition of the threshold fine fraction *F_th_* in terms of specific gravity *G_s_* and void ratio *e*; then threshold fine fraction *F_th_* is the mass fraction of fines divided by total mass *M_Total_* when the pore spaces between coarse grains are completely filled with fines (See detailed derivation in [22]):

(1) $$F_{th}=\frac{M_{F}}{M_{\mathrm{Total}}}=\frac{M_{F}}{M_{F}+M_{C}}=\frac{e_{C}}{1+e_{C}+e_{F}},$$

where *M* indicates the mass, and the subscripts *C* and *F* indicate coarse and fine grains, respectively. The threshold fines fraction implies that there are two threshold values. The low-threshold fines fraction *F_th_*|*^L^* is obtained when coarse grains are densely packed and fine grains are loosely packed:

(2) $${\left.F_{\mathrm{th}}\right|}^{L}=\frac{e_{C}{}^{\min}}{1+e_{C}{}^{\min}+e_{F}{}^{\max}}.$$

By contrast, the high-threshold fines fraction *F_th_*|*^H^* is achieved when coarse grains are loosely packed and fine grains are densely packed:

(3) $${\left.F_{\mathrm{th}}\right|}^{H}=\frac{e_{C}{}^{\max}}{1+e_{C}{}^{\max}+e_{F}{}^{\min}}.$$

Clearly, the low- and high-threshold fines fractions divide the microstructure of binary mixtures into three groups: (1) Coarse-dominant at fines fraction *F_F_* < *F_F_*|*^L^*, (2) transitional at fine fraction *F_F_*|*^L^* < *F_F_* < *F_F_*|*^H^*, and (3) fine-dominant mixtures at fines fraction *F_F_* > *F_F_*|*^H^*. However, the packing of finer grains in the pore spaces between coarse grains is different from a packing condition in a bulk scaled-soil matrix, although the physics-inspired threshold fractions conceptually divide soil into three categories. Therefore, the data-based correction factor *α* is used to define the data-driven high-threshold fines fraction for coarse–fine mixtures:

(4) $${\left.F_{\mathrm{th}}\right|}^{H*}=\frac{\alpha \cdot e_{C}{}^{\max}}{1+\alpha \cdot e_{C}{}^{\max}+e_{F}{}^{\min}},$$

where the data-based correction factor is *α* = 1.3 for fine-coarse mixtures and *α* = 2.5 for sand-gravel mixtures.

### 2.2. Void Ratios: Correlations from Soil Index Properties

The packing conditions of gravels and/or sands are not highly sensitive to effective stress in comparison to fines such as silts and clays. The particle shape such as roundness *R* and coefficient of uniformity *C_u_* for gravels and sands seem to be more relevant in the determination of maximum void ratio *e^max^* and minimum void ratio *e^min^* [3,26]:

(5) $$e^{\max}=0.032+\frac{0.154}{R}+\frac{0.522}{C_{u}}$$

(6) $$e^{\min}=-0.012+\frac{0.082}{R}+\frac{0.371}{C_{u}}.$$

The maximum and minimum void ratios *e^max^* and *e^min^* represent feasible loose and dense packing conditions, respectively. Note that roundness is *R* = (∑*r_i_*/*N*)/r_*max*_ where *r_i_* = radius of curvature for each inscribed sphere, *N* = number of inscribed spheres, and r_*max*_ = maximum radius of the inscribed sphere.

By contrast, the RSCS adopts K_o_-compression line and uses two-reference void ratios for fines: (1) Void ratio at 10 kPa (=*e_F_|^*10kPa*^*) for soft and (2) void ratio at 1 MPa (=*e_F_|^*1MPa*^*) for stiff packing conditions. The two void ratios for fines can be a function of liquid limit *LL* from the correlations suggested by previous studies [27,28]:

(7) $${\left.e_{F}\right|}^{10\mathrm{kPa}}=0.0266\mathrm{LL}+0.07$$

(8) $${\left.e_{F}\right|}^{1\mathrm{MPa}}=0.011\mathrm{LL}+0.21.$$

Note that the use of Equations (7) and (8) requires the liquid limit obtained from the fines passing through Sieve No. 200.

## 3. Data Compilation

This section examines the changes in engineering soil properties complied from the literature as a function of fines fraction *F_F_* in the context of low- and high-threshold fines fractions using Equations (2) and (4). Collected soil properties include void ratio, shear wave velocity, compression index, friction angle, and hydraulic conductivity.

### 3.1. Void Ratio

**Figure 1:**
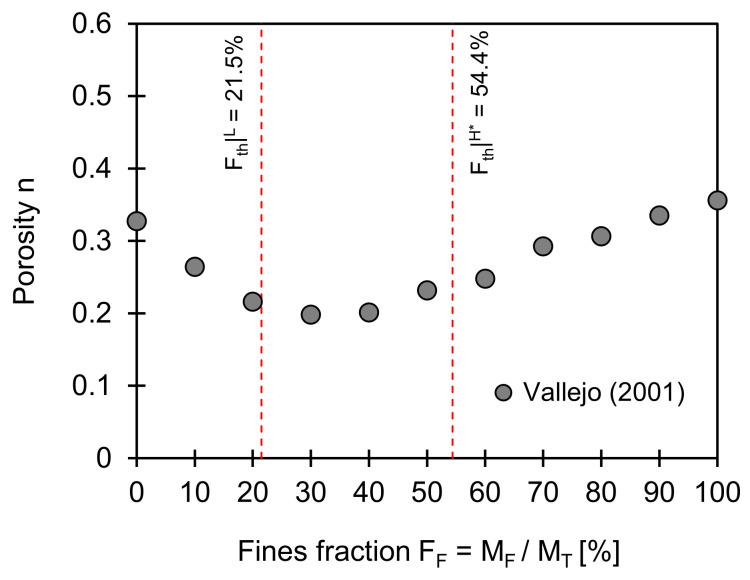
Porosity versus fines fraction. Two red dotted lines indicate low and data-adjusted high-threshold fines fractions computed using Equations (2) and (4). Input parameters used for estimation of two boundaries are *e_C_^max^* = 0.805, *e_C_^min^* = 0.533, *e_F_^max^* = 0.949; *e_F_^min^* = 0.689, and *F_th_*|*^L^* = 21.5%, and *F_th_*|^H^* = 54.4%. Note: Data extracted from Vallejo [30].

Figure 1 shows the changes in porosity against the fines fraction in a coarse–fine mixture. Note that porosity refers to the ratio of pore volume to total volume, while void ratio denotes that of pore volume to soil (i.e., solid) volume. The two red dotted lines superimposed in Figure 1 indicate the low- and data-adjusted high-threshold fractions computed from Equations (2) and (4) (input used for computations: *e_C_^max^* = 0.805, *e_C_^min^* = 0.533, *e_F_^min^* = 0.949; *e_F_^min^* = 0.689, *F_th_*|*^L^* = 21.5%, and *F_th_*|^H^* = 54.4%). A bilinear trend characterizes the change in porosity as a function of the fines fraction. The porosity decreases with the fines fraction below the low threshold value, whereas the porosity begins to increase when the fines fraction exceeds the high-threshold value. The increase in porosity above the high-threshold fines fraction may be more pronounced as fines attain a higher specific surface [29]. Most notably, there is a transitional turning point in porosity within the two theoretically predicted threshold fines fractions.

### 3.2. Shear Wave Velocity

**Figure 2:**
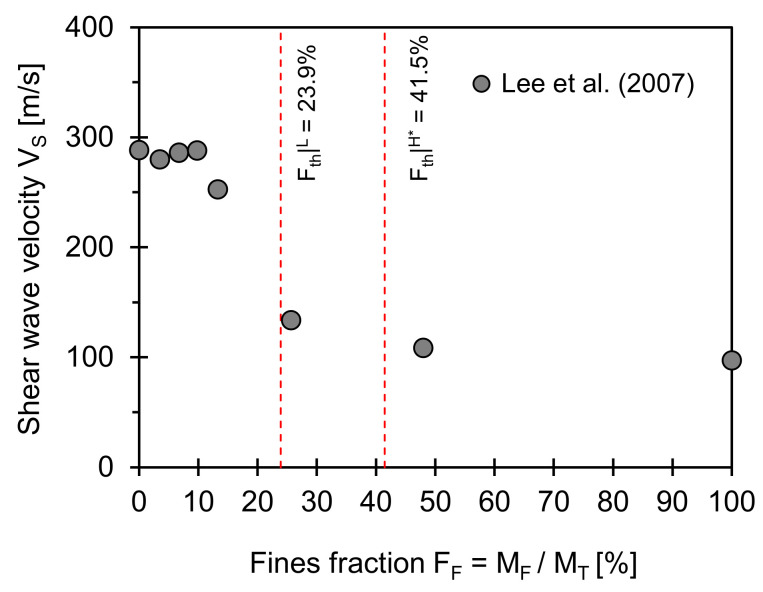
Shear wave velocity versus fines fraction. Two red dotted lines indicate low and data-adjusted high-threshold fines fractions computed using Equations (2) and (4). Input parameters used for estimation of two boundaries are *e_C_^max^* = 0.85, *e_C_^min^* = 0.59, *e_F_^max^* = 0.88; *e_F_^min^* = 0.56, and *F_th_*|*^L^* = 23.9%, and *F_th_*|^H^* = 41.5%. Note: Data extracted from Lee et al. [21].

Figure 2 presents the shear wave velocity for a coarse–fine mixture versus fine fraction. The two red dotted lines superimposed in Figure 2 correspond to the low- and data-adjusted high-threshold fractions computed from Equations (2) and (4) (input used for computations: *e_C_^max^* = 0.850, *e_C_^min^* = 0.590, *e_F_^min^* = 0.879; *e_F_^min^* = 0.560, *F_th_*|*^L^* = 23.9%, and *F_th_*|^H^* = 41.5%). Finer grains seem to reinforce a load-carrying skeleton developed by coarse grains when the fines fraction is below the low threshold value. However, there is a dramatic change in shear wave velocity in the transitional zone. At a fines fraction above the high-threshold value, fines play a primary role in small strain wave propagation.

### 3.3. Compression Index

**Figure 3:**
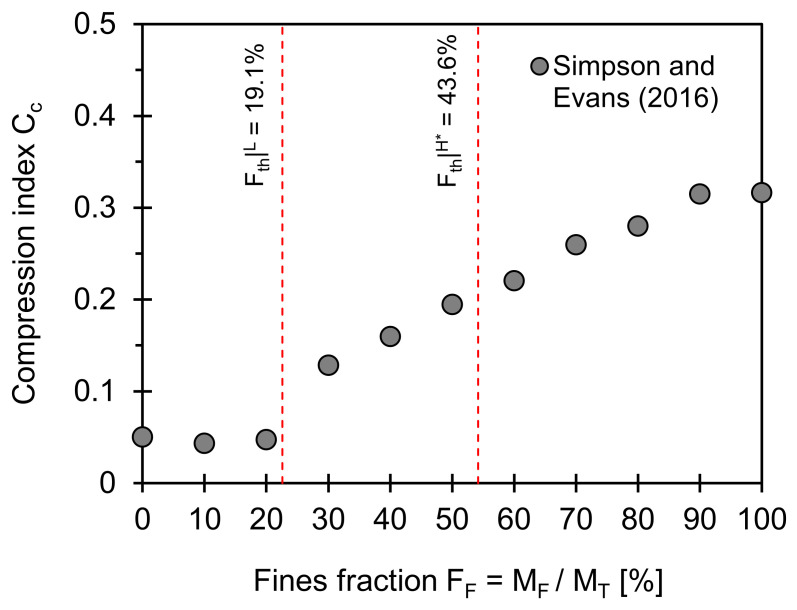
Compression index versus fines fraction. Two red dotted lines indicate low and data-adjusted high-threshold fines fractions computed using Equations (2) and (4). Input parameters used for estimation of two boundaries are *e_C_^max^* = 0.912, *e_C_^min^* = 0.584, *e_F_^max^* = 1.47; *e_F_^min^* = 0.53, and *F_th_*|*^L^* = 19.1%, and *F_th_*|^H^* = 43.6%. Note: Data extracted from Simpson and Evans [31].

Figure 3 plots the compression index of a coarse–fine mixture as a function of fines fraction. Note that compression index is a slope of the graph between void ratio and effective stress in log-scale, and it is used to estimate the amount of settlement in normally consolidated soils. Figure 3 also shows the low and data-adjusted high-threshold fractions computed from Equations (2) and (4). The low and high-threshold fines fractions are between 6.0% and 31.1% in this case (input used for computations: *e_C_^max^* = 0.912, *e_C_^min^* = 0.584, *e_F_^min^* = 1.47; *e_F_^min^* = 0.534, *F_th_*|*^L^* = 19.1%, and *F_th_*|^H^* = 43.6%). There is a bilinear trend in the compression index versus fines fraction; the two threshold values successfully capture the transitional behavior in the compression index. Note that the fines fraction corresponding to the transition becomes small at a high liquid limit [22].

### 3.4. Friction Angle

**Figure 4:**
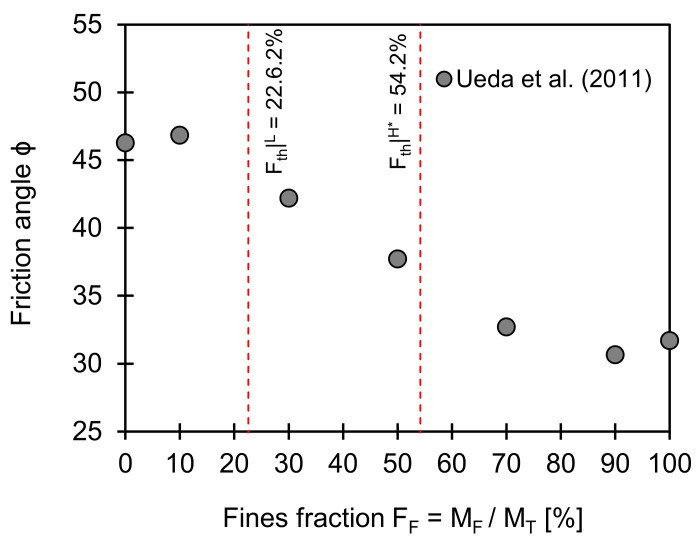
Friction angle versus fines fraction. Two red dotted lines indicate low and data-adjusted high-threshold fines fractions computed using Equations (2) and (4). Input parameters used for estimation of two boundaries are *e_C_^max^* = 0.737, *e_C_^min^* = 0.548, *e_F_^max^* = 0.879; *e_F_^min^* = 0.56, and *F_th_*|*^L^* = 22.6%, and *F_th_*|^H^* = 54.2%. Note: Data extracted from Ueda et al. [32].

Figure 4 shows the friction angles for a coarse–fine mixture plotted against the fines fraction. Note that friction angle is one of the strength parameters that describe the ability of soils to resist shear stress. The low- and data-adjusted high-threshold fractions are computed from Equations (2) and (4) and overlapped on Figure 4 (input used for computations: *e_C_^max^* = 0.737, *e_C_^min^* = 0.548, *e_F_^min^* = 0.879; *e_F_^min^* = 0.56, *F_th_*|*^L^* = 22.6%, and *F_th_*|^H^* = 54.2%). The change in friction angle with the fines fraction depends on whether fines occupy the pores between coarse grains (=reinforcing effect) or separate the contacts between coarse grains (=disturbance effect) followed by coarse grains floating in the soil matrix. A gradual decrease in friction angle with the fines fraction occurs between the low- and high-threshold fines fraction. This observation indicates that either coarse or fine grains (or both grains) may control the normal stress-dependent energy loss in transitional granular mixtures. 

### 3.5. Hydraulic Conductivity 

**Figure 5:**
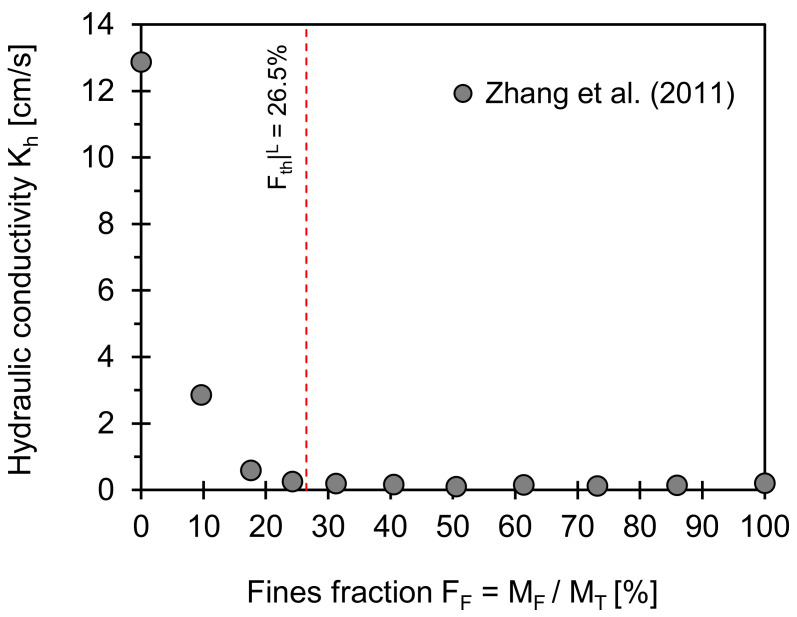
Hydraulic conductivity versus fines fraction. Red dotted lines indicate low threshold fines fraction computed using Equation (2). Input parameters used for estimation of two boundaries are *e_C_^min^* = 0.45 and *e_F_^max^* = 1.51, and *F_th_*|*^L^* = 26.5%. Note: Data extracted from Zhang et al. [33].

Figure 5 plots the hydraulic conductivities for a coarse–fine mixture versus the fines fraction. Note that hydraulic conductivity presents the capacity of porous media to transmit fluid through pore spaces. The low-threshold fines fraction computed from Equation (2) is superimposed on Figure 5. There is a dramatic change in hydraulic conductivity when the fines fraction drops below the low-threshold value. This observation indicates that even small amounts of fines play a critical role in the characteristic pore size that determines the fluid flow. Furthermore, the data-adjusted high-threshold fines fraction may be more relevant for mechanical properties such as soil stiffness and strength rather than hydraulic conduction phenomena. Fines with a higher specific surface will lead to a lower threshold fines fraction.

## 4. Materials and Methods

### 4.1. Savannah River Site

The Savannah River Site (SRS) is located approximately 25 miles south of Augusta, Georgia. SRS was constructed for the production and storage of nuclear materials. Although the stability of SRS was critical owing to the use of nuclear energy, the dissolution of old calcareous sediments known as the Santee Formation was discovered at the depth of 30–45 m after the initial construction of SRS after World War II [34,35]. The sediments at SRS have been characterized in terms of voids owing to the dissolution, caves caused by complicated geologic process, and karst-like characteristics [35]. Geological investigations revealed that the upper part of the SRS (< 25 m) consists of very old and layered deposits of sands, clayey sands, and silty sands, which have a complex stress history, while the sand in the lower part of SRS (> 25 m) contain carbonate mineral [35].

### 4.2. Savannah River Soil: Specimen Preparation 

**Figure 6:**
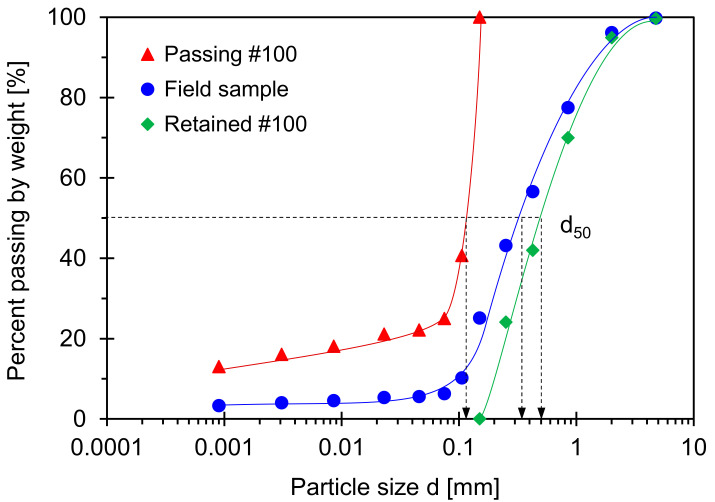
Particle size distribution of Savannah River sand (boring number: HPC-1, Sampling depth: 19.20 m–19.35 m).

This experimental program prepares three specimens with Savannah River sand (Boring number: HPC-1; Sampling depth: 19.20–19.35 m): (1) Original field sample, (2) fine-grained soil passing through Sieve No. 100 (corresponding particle size = 0.15 mm), and (3) coarse-grained soil retained on Sieve No. 100. Figure 6 shows the particle size distribution curve for three specimens. Table 1 summarizes the soil index properties for all specimens. The original field sample is classified as poorly graded sand with clay, i.e., SP-SC, according to the Unified Soil Classification System (USCS) [6]. The liquid limit LL for the original field sample is LL = 31.3 obtained using soil fraction passing Sieve No. 40 while the LL is 60.1 obtained using soil fraction passing Sieve No. 200. The preparation of the three specimens starts with pastes mixed with water. The gravimetric water contents *ω* for the three pastes are greater than the liquid limit *LL* for each sample to ensure fully saturated conditions (i.e., *ω* ≈ 1.1 × *LL*). The well-controlled gravimetric water contents lead to very loose or soft specimens when placed in an oedometer cell for a vertical loading procedure.

### 4.3. Instrumented Oedometer Cell

**Figure 7:**
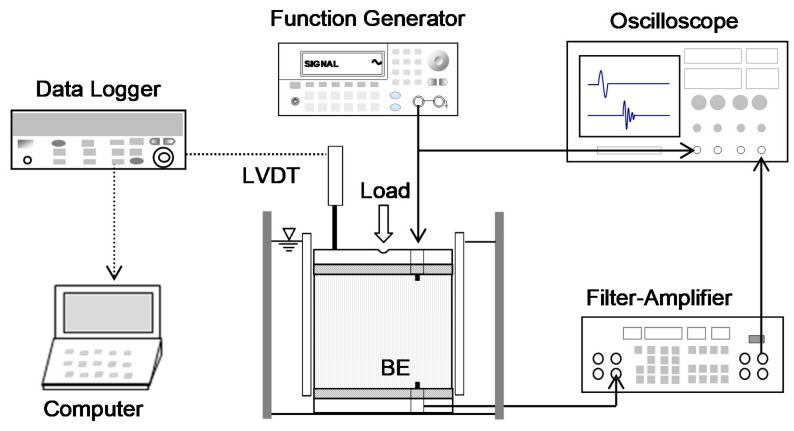
Floating oedometer cell with bender elements and peripheral devices for measurement of shear waves (note: BE denotes bender element).

This study uses a modified floating oedometer cell to investigate the evolution of the void ratio, compressibility, and shear wave velocity for each loading step as the vertical effective stress increases (Figure 7). Dimensions of the floating ring are 73 mm and 50 mm in inner diameter and height, respectively. The floating ring is a very efficient design to reduce the side friction effect between the wall and soil particles. Top and bottom caps include bender elements that function as shear wave transducers, mounted with epoxy in the nylon screws. The procedure of the odometer test follows a typical consolidation test. The loading procedure for the vertical effective stress applied on the specimen consists of 10 stages from 3 to 1154 kPa. For the unloading step, the vertical stress is reduced in reverse order.

### 4.4. Deformation and Shear Wave Monitoring

The LVDT (TransTek DC 0242, Trans-Tek, Inc., Ellington, CT, USA) connected to a da-ta logger (Keysight 34970A, Keysight Technologies, Santa Rosa, CA, USA) and computer, as shown in Figure 7, continuously monitors the vertical deformation of specimens during the loading procedure. This study selects a parallel type of bender elements to generate and detect shear waves and minimize the effect of crosstalk [36]. The dimensions of bender elements are 12.7 × 8.0 × 0.7 (length × width × thickness in mm) with a 5.0-mm cantilever length. The function generator forms step input signals with a 10-V input voltage (Keysight 33210A, Keysight Technologies, Santa Rosa, CA, USA), and elastic shear waves transformed by the bender element then propagate through the soils. The filter ampli-fier (Krohn-Hite 3364, Krohn-Hite Corporation, Brockton, MA, USA) uses 500 Hz and 200 kHz for high- and low-pass filtering, respectively. The computer saves the signals shown on the oscilloscope (Keysight DSOX 2014A). The minimum sampling frequency of received signals is 1 MHz. The number of signals stacked for a high signal-to-noise ratio is 1024 [37]. The shear wave signals are saved at the end of each loading stage when the deformation reaches an asymptote.

## 5. Results and Discussion

### 5.1. Vertical Deformation and Void Ratio

**Figure 8:**
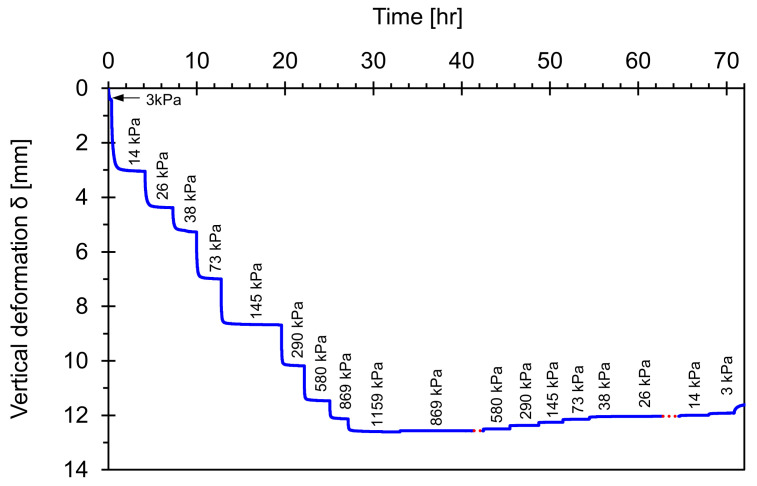
Load-deformation response for original field sample under zero-later strain conditions.

Figure 8 shows the changes in vertical deformation for the original field sample during the loading and unloading history. The vertical deformation increases during the double increment loading process; however, there seems to be significant locked-in horizontal stress during unloading. Once again, this study uses this deformation data to determine the end of each loading step when the deformation reaches an asymptotic trend. Furthermore, this well-controlled load-deformation response provides a typical soil compaction trend in the void ratio versus vertical effective stress space to facilitate further analyses.

**Figure 9:**
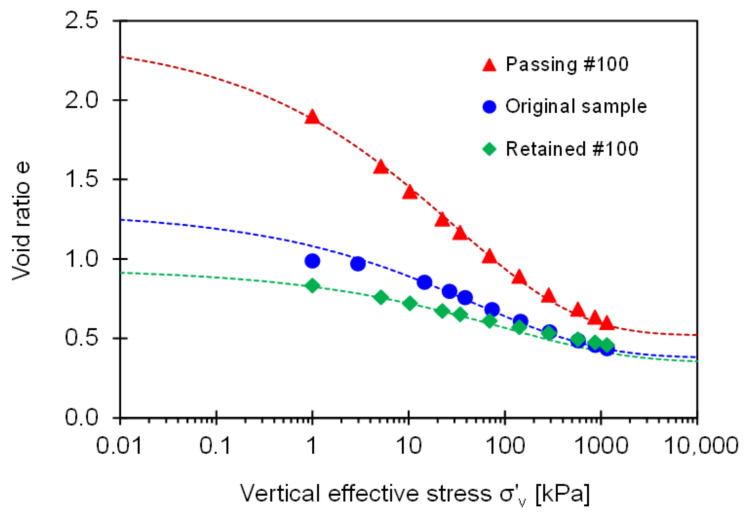
Compaction curves for three tested specimens in view of void ratio *e* against vertical effective stress *σ*′*_v_*.

In this context, Figure 9 presents the void ratio versus vertical effective stress for the original field sample, fine-grained soil passing through Sieve No. 100, and coarse-grained soil retained on Sieve No. 100. The void ratio for all specimens decreases with the effective stress. Clearly, there exists an asymptotic compaction trend at very low and high effective stress levels.

However, the classical compaction model, i.e., *e* = *e_1 kPa_* − *C_c_*∙log[*σ*′*_z_*/1 kPa], where *C_c_* is compression index, may not capture the two asymptotic void ratios *e_L_* at very low effective stress (i.e., *σ*′*_z_*→0) and *e_H_* at very high effective stress (i.e., *σ*′*_z_*→∞). Therefore, this study uses an asymptotically correct exponential compressibility model to fit the evolution of void ratio *e* as a function of vertical effective stress *σ*′*_z_* [28]:

(9) $$e=e_{H}+\left(e_{L}-e_{H}\right)\exp\left[-{\left(\frac{\sigma_{z}^{\prime }}{\sigma_{c}^{\prime }}\right)}^{\eta }\right]$$

where *σ*′*_c_* is the characteristic effective stress, and *η* is a model parameter. We test different values of model parameter *η* and select *η* = 1/3 that result in the best prediction (see related implications in [38,39,40]). There is a distinct difference in the constitutive model parameters across the three specimens tested in this study (fine-grained soil passing through Sieve No. 100: *e_L_* = 2.4, *e_H_* = 0.52, and *σ*′*_c_* = 30 kPa; original field sample: *e_L_* = 1.3, *e_H_* = 0.38, *σ*′*_c_* = 50 kPa; and coarse-grained soil retained on Sieve No. 100: *e_L_* = 0.94, *e_H_* = 0.35, *σ*′*_c_* = 100 kPa).

Regarding threshold fines fractions, this study considers the asymptotic void ratio *e_L_* for loose packing conditions and *e_H_* for dense packing conditions. Then, the low- and high-threshold fines fractions computed from Equations (2) and (4) correspond to *F_th_*|*^L^* = 9.3% and *F_th_*|^H^* = 44.3%, where the input parameters are *e_C_^max^* = 0.94, *e_C_^min^* = 0.35, *e_F_^max^* = 2.4, and *e_F_^min^* = 0.52. This analysis indicates that the fines fraction *F_F_* ≈ 10% in the original field sample is slightly higher than the low-threshold fines fraction *F_th_*|*^L^*; consequently, the RSCS classifies the original field sample as a transitional mixture. Thus, either fine, coarse, or both fractions could be responsible for the mechanical response. Further analyses associated with transitional compressible behavior follow in the next section.

### 5.2. Compressibility at Each Loading Step (Large Strain)

**Figure 10:**
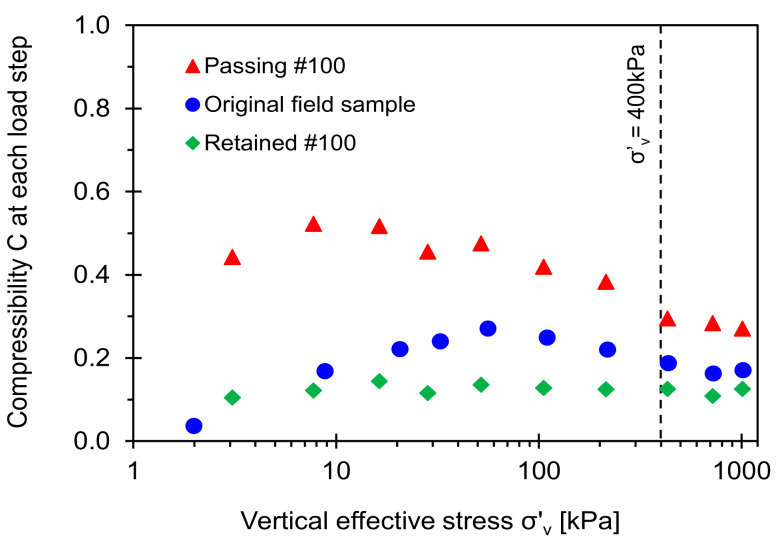
Compressibility for Savannah River sand estimated at consecutive loading steps versus vertical effective stress (note: Sampling depth is 19.20 m–19.35 m).

The load-deformation response is not linear, as shown in the semi-log *e* vs. *σ*′*_v_* space (Figure 9). Therefore, this study emphasizes the change in the void ratio between two consecutive loading steps in Figure 9 that defines the compressibility *C* = (*e_i_* − *e_i + 1_*)/[log(*σ_i + 1_*/*σ_i_*)] between *i* and *i* + 1 loading steps. Figure 10 plots the slop *C* computed at each loading step as a function of the vertical effective stress. The evolution of the compressibility slope for the original field sample indicates that either coarse, fine, or both grains are likely to contribute to the large strain stiffness at effective stress *σ*′*_v_* < 400 kPa. In particular, the low fines fraction in the original field sample (i.e., *F_F_* = 10%) plays a critical role in determining large strain stiffness in the soil classified as a transitional mixture. The slope *C* for the original field sample reaches an asymptotic trend when the effective stress exceeds *σ*′*_v_* ≈ 400 kPa that may match with an in-situ overburden pressure (note: Sampling depth is 19.20–19.35 m, See similar cases in Yoon et al. [41]).

### 5.3. Shear Wave Velocity (Small Strain)

**Figure 11:**
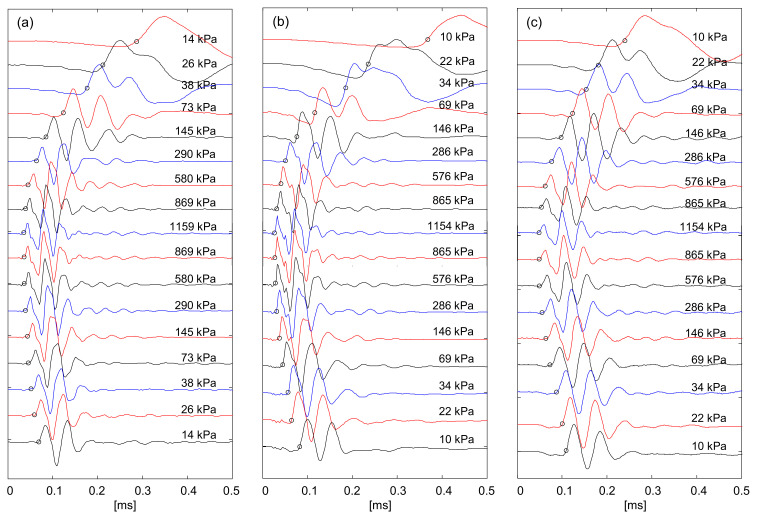
Shear wave signals for Savannah River sands during K_o_ loading and unloading: (**a**) Original field sample; (**b**) Fine-grained soil passed through No. 100 sieve; (**c**) Coarse-grained soil retained on No. 100 sieve.

Figure 11 displays the shear wave signatures measured for the three specimens during K_o_ loading and unloading.

**Figure 12:**
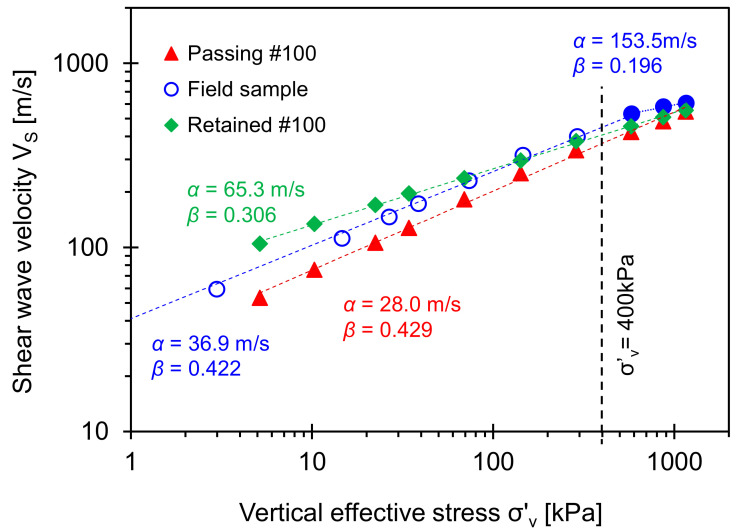
Shear wave velocity for Savannah River sands versus vertical effective stress (note: Sampling depth is 19.20 m–19.35 m).

The time to the first arrival for shear waves is determined by considering the near-field effect [36]. The first arrival time for all specimens decreases with the effective stress. Then, the tip-to-tip distance *L* for the bender elements and first arrival time *t* provide the shear wave velocity *Vs* = *L*/*t*. The shear wave velocity follows a power function of effective stress [5,42]:

(10) $$V_{S}=\alpha \;{\left(\frac{\sigma \prime_{v}}{1\;\mathrm{kPa}}\right)}^{\beta }$$

where *α*-factor [m/s] is the shear wave velocity at *σ*′*_v_* = 1 kPa, and *β*-exponent reflects the sensitivity of the shear wave velocity to the vertical effective stress. Figure 12 shows the shear wave velocity versus vertical effective stress during loading.

The *α*-factor and *β*-exponent for the original field sample obtained at *σ*′*_v_* < 400 kPa is consistent with the *α* and *β* values for clayey soils, while the *α*-factor and *β*-exponent for original field sample at *σ*′*_v_* > 400 kPa belong to the typical sandy soil group (see reported data in [43]). The *β*-exponent for the original field sample obtained at *σ*′*_v_* < 400 kPa is slightly smaller than that of the fine-grained soil passing Sieve No. 100. However, there is a clear transition of the *β*-exponent when the effective stress exceeds *σ*′*_v_* = 400 kPa (i.e., *β* = 0.422 to 0.196). This observation is consistent with the asymptotic slope in Figure 10 at *σ*′*_v_* > 400 kPa. The bilinear trend in *α* and *β* values for the original field sample indicates that the specimen may retain the memory of the in-situ overburden pressure (note: Sampling depth is 19.20 m–19.35 m). In particular, the crossing of the shear wave velocity trends indicates that either fine, coarse, or both fractions will determine the mechanical properties in transitional mixtures despite a 10% fines fraction in the original field sample.

### 5.4. Fines Migration and Clogging

**Figure 13:**
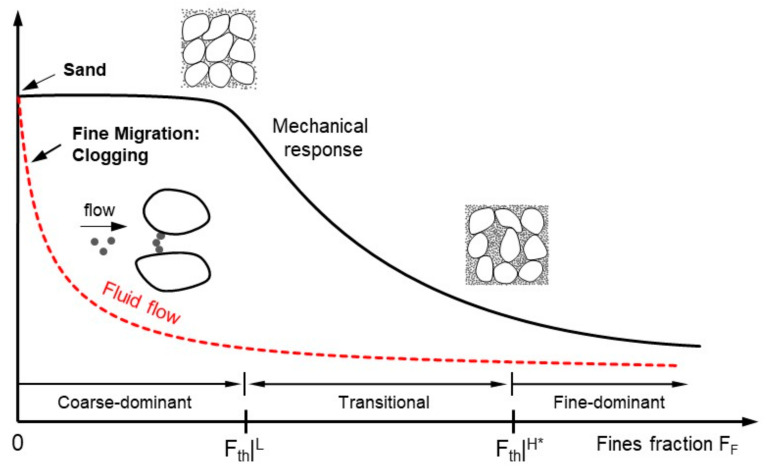
Conceptual drawing of changes in mechanical response and fluid flow in terms of low and data-adjusted high-threshold fines fractions.

The data compilation and experimental results analyzed in the context of low and data-adjusted high fines fractions emphasize the role of fines on the mechanical response in the transitional mixture according to the RSCS. However, the low-threshold fines fraction *F_th_*|*^L^* in Equation (2) may not adequately capture the threshold value with regard to the hydraulic conductivity point because the fluid-flow-driven fines migration through a porous network may lead to the formation of bridges at pore throats, as shown in Figure 13.

Thereafter, a clogging ring might develop near production wells when the fines fraction *F_F_* is much lower than the low threshold fines fraction *F_th_*|*^L^*. Thus, the hydraulic conductivity of mixtures may dramatically decrease at a fines fraction of *F_F_* < *F_th_*|*^L^*. The bridge-induced clogging is affected by the particle shape [44]. The stability of bridges increases as the particle shape becomes rough and less spherical [45]. Therefore, the soil classification system should include particle shape, and considerations are required for the conservative threshold values regarding hydraulic conductivity.

### 5.5. New Features of RSCS

There are common limitations to current classification systems. They adopt classification boundaries fixed for coarse–fine mixtures although fine-grained soils may exhibit a broad range of plasticity. Particle shape affects the packing density of the coarse fraction; however, particle shape does not feature in any systems. The effect of plastic fines on soil engineering properties is not adequately reflected by the 50%. Finally, current classification systems cannot capture the importance of pore-fluid chemistry on fines behavior. A new soil classification system known as the Revised Soil Classification System RSCS is proposed to cover the limitations above-mentioned and to address the physics-inspired and data-driven framework. The soil classification boundaries in RSCS are not fixed (i.e., not 50% for coarse- or fine-grained soils and gravel or sand), but soil-specific that change with soil index properties such as liquid limit, coefficient of uniformity, and particle shape. The RSCS uses a dual-name nomenclature where the first letter(s) identifies the component that controls the mechanical properties and the second letter indicates the component that controls fluid flow. The RSCS requests further fines classification in view of plasticity and electrical sensitivity if the soil group includes fines described as either “F” for mechanical control or “(F)” for fluid flow control. It is time to test the RSCS with a broad range of soils. In this context, this study examines the behavior of the natural soil sample within the RSCS framework.

## 6. Conclusions

The goal of this study is to characterize the engineering behavior of Savannah River sand in terms of the void ratio, compressibility, and small strain stiffness within the framework of the threshold fines fraction. Volumetric-gravimetric packing analyses combined with data define the low- and data-adjusted high-threshold fines fractions. The two threshold values are superimposed on the engineering properties against the fines fractions to test their validity. Oedometer tests and shear wave measurements are conducted using deformation and elastic wave sensors for three specimens with different particle size distributions to investigate the evolution of the soil stiffness at high and low strain levels. A published dataset extracted from the literature and experimental results gathered using electromagnetic- and piezo-crystal-based sensors are analyzed in the context of low and data-adjusted high fines fractions. Analyses highlight the critical role of fines on the engineering properties of sediments when classified as a transitional mixture according to RSCS. Salient conclusions made in this study are as follows:

- Low *F_th_*|*^L^* and data-adjusted high *F_th_*|^H^* threshold fines fractions defined by theoretical volumetric–gravimetric relations successfully capture the transitional behavior in engineering properties (e.g., void ratio, shear wave velocity, compression index, and friction angle).
- The dramatic change in hydraulic conductivity below the low-threshold fines fraction *F_th_*|*^L^* highlights that a small number of fines plays a critical role in the fluid flow (e.g., clogging). Furthermore, the data-adjusted high-threshold fines fractions may be more relevant to mechanical properties than hydraulic conduction phenomena.
- The RSCS classified the Savannah River sand with a fines fraction *F_F_* ≈10% as a transitional mixture (*F_th_*|*^L^* = 9.3%; *F_th_*|^H^* = 44.3%).
- The evolutions of the slope with regard to the compressibility and shear wave velocity of Savannah River sand indicate that either coarse, fine, or both grains are likely to contribute to the high and low strain stiffness at an effective stress *σ*′*_v_* < 400 kPa. The trends of slope change when the effective stress exceeds *σ*′*_v_* ≈ 400 kPa and may match an in-situ overburden pressure.

## Figures and Tables

**Table 1:** Index properties of tested soil.

HPC-1 (19.2~19.35 m)	D_10_ [mm]	D_60_ [mm]	D_50_ [mm]	C_u_	Specific Surface S_s_ [m^2^/g]	Liquid Limit LL	R	PI	USCS	RSCS
Original Field sample	0.1	0.48	0.35	4.8	73.4	60.1	0.53	18.1	SP-SC	CF
Passing through Sieve No. 100	<0.001	0.12	0.12	-	-	-	-	-	-	-
Retained on Sieve No. 100	0.19	0.68	0.56	3.6	-	-	-	-	-	-

Note: C_u_ = coefficient of uniformity, R = roundness, PI = plastic index.

## Data Availability

The data presented in this study are available on request from the corresponding author. The data are not publicly available due to ongoing research project.

## References

[1] Thevanayagam S., Shenthan T., Mohan S., Liang J. (2020). Undrained fragility of clean sands, silty sands, and sandy silts. J. Geotech. Geoenviron. Eng..

[2] Fiès J.C., Bruand A. (1998). Particle packing and organization of the textural porosity in clay–silt–sand mixtures. Eur. J. Soil Sci..

[3] Santamarina J.C., Cho G.C. (2004). Soil behaviour: The role of particle shape. In Advances in geotechnical engineering: The skempton conference. ICE Virtual Libr..

[4] Cho G.C., Dodds J., Santamarina J.C. (2006). Particle shape effects on packing density, stiffness, and strength: Natural and crushed sands. J. Geotech. Geoenviron. Eng..

[5] Santamarina J.C., Klein K.A., Fam M.A. (2001). Soils and Waves—Particulate Materials Behavior, Characterization and Process Monitoring.

[6] ASTM (2011). ASTM D2487. Standard Practice for Classification of Soils for Engineering Purposes (Unified Soil Classification System).

[7] Lade P.V., Yamamuro J.A. (1997). Effects of nonplastic fines on static liquefaction of sands. Can. Geotech. J..

[8] Salgado R., Bandini P., Karim A. (2000). Shear strength and stiffness of silty sand. J. Geotech. Geoenviron. Eng..

[9] Carraro J.A.H., Prezzi M., Salgado R. (2009). Shear strength and stiffness of sands containing plastic or nonplastic fines. J. Geotech. Geoenviron. Eng..

[10] Thevanayagam S. (1998). Effect of fines and confining stress on undrained shear strength of silty sands. J. Geotech. Geoenviron. Eng..

[11] Yang S., Lacasse S., Sandven R. (2006). Determination of the transitional fines content of mixtures of sand and non-plastic fines. Geotech. Test. J..

[12] Jung J.W., Jang J., Santamarina J.C., Tsouris C., Phelps T.J., Rawn C.J. (2012). Gas production from hydrate-bearing sediments: The role of fine particles. Energy Fuels.

[13] Choo H., Burns S.E. (2015). Shear wave velocity of granular mixtures of silica particles as a function of finer fraction, size ratios and void ratios. Granul. Matter.

[14] Lee J.S., Dodds J., Santamarina J.C. (2007). Behavior of rigid-soft particle mixtures. J. Mater. Civ..

[15] Lee C., Truong Q.H., Lee W., Lee J.S. (2010). Characteristics of rubber-sand particle mixtures according to size ratio. J. Mater. Civ..

[16] Kim S.Y., Park J., Lee J.S. (2021). Coarse-fine mixtures subjected to repetitive Ko loading: Effects of fines fraction, particle shape, and size ratio. Powder Technol..

[17] Kenney T.C. (1977). Residual strengths of mineral mixtures. Proceedings of the 9th International Conference on Soil Mechanics and Foundation Engineering, Tokyo, Japan, 10–15 July 1997.

[18] Indrawan I.G.B., Rahardjo H., Leong E.C. (2006). Effects of coarse-grained materials on properties of residual soil. Eng. Geol..

[19] Monkul M.M., Ozden G. (2007). Compressional behavior of clayey sand and transition fines content. Eng. Geol..

[20] Tiwari B., Ajmera B. (2011). Consolidation and swelling behavior of major clay minerals and their mixtures. Appl. Clay Sci..

[21] Lee J.S., Guimaraes M., Santamarina J.C. (2007). Micaceous sands: Microscale mechanisms and macroscale response. J. Geotech. Geoenviron. Eng..

[22] Park J., Santamarina J.C. (2017). Revised soil classification system for coarse-fine mixtures. J. Geotech. Geoenviron. Eng..

[23] Park J., Castro G.M., Santamarina J.C. (2018). Closure to “Revised soil classification system for coarse-fine mixtures” by Junghee Park and J. Carlos Santamarina. J. Geotech. Geoenviron. Eng..

[24] Fraser H.J. (1935). Experimental study of the porosity and permeability of clastic sediments. J. Geol..

[25] Radjai F., Wolf D.E., Jean M., Moreau J.J. (1998). Bimodal character of stress transmission in granular packings. Phys. Rev. Lett..

[26] Youd T.L. (1973). Factors Controlling Maximum and Minimum Densities of Sands, Evaluation of Relative Density and Its Role in Geotechnical Projects Involving Cohesionless Soils.

[27] Skempton A.W. (1944). Notes on compressibility of clays. J. Geol. Soc..

[28] Chong S.H., Santamarina J.C. (2016). Soil compressibility models for a wide stress range. J. Geotech. Geoenviron. Eng..

[29] Mitchell J.K., Soga K. (2005). Fundamentals of Soil Behavior.

[30] Vallejo L.E. (2001). Interpretation of the limits in shear strength in binary granular mixtures. Can. Geotech. J..

[31] Simpson D.C., Evans T.M. (2016). Behavioral thresholds in mixtures of sand and kaolinite clay. J. Geotech. Geoenviron. Eng..

[32] Ueda T., Matsushima T., Yamada Y. (2011). Effect of particle size ratio and volume fraction on shear strength of binary granular mixture. Granul. Matter.

[33] Zhang Z.F., Ward A.L. (2011). Determining the porosity and saturated hydraulic conductivity of binary mixtures. Vadose Zone J..

[34] Larrahondo-Cruz J.M. (2011). Carbonate Diagenesis and Chemical Weathering in the Southeastern United States: Some Implications on Geotechnical Behavior. Ph.D. Thesis.

[35] Ku T. (2012). Geostatic Stress State Evaluation by Directional Shear Wave Velocities, with Application towards Geocharacterization at Aiken, SC. Ph.D. Thesis.

[36] Lee J.S., Santamarina J.C. (2005). Bender elements: Performance and signal interpretation. J. Geotech. Geoenviron. Eng..

[37] Santamarina J.C., Fratta D. (2005). Discrete Signals and Inverse Problems: An Introduction for Engineers and Scientists.

[38] Terzariol M., Park J., Castro G.M., Santamarina J.C. (2020). Methane hydrate-bearing sediments: Pore habit and implications. Mar. Pet. Geol..

[39] Park J., Santamarina J.C. (2020). The critical role of pore size on depth-dependent microbial cell counts in sediments. Sci. Rep..

[40] Lyu C., Park J., Carlos Santamarina J. (2021). Depth-dependent seabed properties: Geoacoustic assessment. J. Geotech. Geoenviron. Eng..

[41] Yoon H.K., Lee C., Kim H.K., Lee J.S. (2011). Evaluation of preconsolidation stress by shear wave velocity. Smart Struct. Syst..

[42] Roesler S.K. (1979). Anisotropic shear modulus due to stress anisotropy. J. Geotech. Eng..

[43] Cha M., Santamarina J.C., Kim H.S., Cho G.C. (2014). Small-strain stiffness, shear-wave velocity, and soil compressibility. J. Geotech. Geoenviron. Eng..

[44] Valdes J.R., Santamarina J.C. (2006). Particle clogging in radial flow: Microscale mechanisms. Soc. Pet. Eng. J..

[45] Valdes J.R., Santamarina J.C. (2008). Clogging: Bridge formation and vibration-based destabilization. Can. Geotech. J..

